# Co-imaging of the tumor oxygenation and metabolism using electron paramagnetic resonance imaging and 13-C hyperpolarized magnetic resonance imaging before and after irradiation

**DOI:** 10.18632/oncotarget.25317

**Published:** 2018-05-18

**Authors:** Masayuki Matsuo, Tatsuya Kawai, Shun Kishimoto, Keita Saito, Jeeva Munasinghe, Nallathamby Devasahayam, James B. Mitchell, Murali C. Krishna

**Affiliations:** ^1^ Radiation Biology Branch, Center for Cancer research, National Cancer Institute, National Institutes of Health, Bethesda, MD, USA; ^2^ Radiation Oncology Branch, Center for Cancer research, National Cancer Institute, National Institutes of Health, Bethesda, MD, USA; ^3^ MRI Research Facility, National Institute of Neurological Disorders and Stroke, National Institutes of Health, Bethesda, MD, USA; ^4^ Department of Radiology, Gifu University Graduate School of Medicine, Gifu City, Japan

**Keywords:** pO_2_, ESR imaging, 1-13C MRI, glycolytic metabolism, radiation therapy

## Abstract

To examine the relationship between local oxygen partial pressure and energy metabolism in the tumor, electron paramagnetic resonance imaging (EPRI) and magnetic resonance imaging (MRI) with hyperpolarized [1-^13^C] pyruvate were performed.

SCCVII and HT29 solid tumors implanted in the mouse leg were imaged by EPRI using OX063, a paramagnetic probe and ^13^C-MRI using hyperpolarized [1-^13^C] pyruvate. Local partial oxygen pressure and pyruvate metabolism in the two tumor implants were examined. The effect of a single dose of 5-Gy irradiation on the pO_2_ and metabolism was also investigated by sequential imaging of EPRI and ^13^C-MRI in HT29 tumors.

A phantom study using tubes filled with different concentration of [1-^13^C] pyruvate, [1-^13^C] lactate, and OX063 at different levels of oxygen confirmed the validity of this sequential imaging of EPRI and hyperpolarized ^13^C-MRI. *In vivo* studies revealed SCCVII tumor had a significantly larger hypoxic fraction (pO_2_ < 8 mmHg) compared to HT29 tumor. The flux of pyruvate-to-lactate conversion was also higher in SCCVII than HT29. The lactate-to-pyruvate ratio in hypoxic regions (pO_2_ < 8 mmHg) 24 hours after 5-Gy irradiation was significantly higher than those without irradiation (0.76 vs. 0.36) in HT29 tumor. The *in vitro* study showed an increase in extracellular acidification rate after irradiation.

In conclusion, co-imaging of pO_2_ and pyruvate-to-lactate conversion kinetics successfully showed the local metabolic changes especially in hypoxic area induced by radiation therapy.

## INTRODUCTION

Tissue partial pressure of oxygen (pO_2_) is linked to many pathophysiological conditions (e.g., ischemic diseases, reperfusion injury, and oxygen toxicity). Oxygen deficiency or hypoxia can increase the tumor's resistance toward cancer treatments, including radiation therapy and chemotherapy [1, 2]. Additionally, hypoxic microenvironments in tumors are known to promote processes driving malignant progression, such as neo-angiogenesis, genetic instability, and metastasis [3–5]. There is a clear oxygen dependent relationship between the radiation dose delivered locally to the tumor and the response to the radiation. This finding was essentially attributed to hypoxic cells being resistant to radiation [6]. Considering the fact that many solid tumors outgrow the blood supply and, therefore, have some regions with chronic and intermittent hypoxia [7–9], investigation of both tumor oxygenation and energy metabolism simultaneously and non-invasively is required to not only understand the radiosensitivity in individual tumor but also assess the treatment efficacy of radiation therapy.

Electron paramagnetic resonance imaging (EPRI) is a spectroscopic technique similar to nuclear magnetic resonance. EPR detects paramagnetic species that have unpaired electrons such as transition metal complexes and free radicals. With the recent availability of trityl radical probes as *in vivo* compatible paramagnetic probes, EPRI is now being explored for mapping tissue oxygen in live animals [8–12]. Recently dynamic nuclear polarization (DNP) techniques which can polarize the nuclear spin of ^13^C-labeled substrates far beyond thermal equilibrium conditions have been devised enabling metabolic MRI [13, 14] to monitor specific enzymatic reactions elevated in cancer. Many studies have shown that the polarization of [1-^13^C] pyruvate provides sufficient MR signal for high spatial and temporal resolution spectroscopy to monitor its metabolites such as lactate catalyzed by the enzyme lactate dehydrogenase (LDH) which is elevated in cancers [15–18]. A clinical study of DNP-MRI using hyperpolarized [1-^13^C]-pyruvate has already been demonstrated in prostate cancer [19, 20] and myocardium [21].

In the present study, non-invasive sequential imaging of EPRI for tumor pO_2_ and DNP-MRI using hyperpolarized [1-^13^C] pyruvate for metabolic profile was conducted to evaluate the pixel by pixel correlation between intratumor pO_2_ and glycolytic profile. The relationship between energy metabolism and tumor oxygen status was investigated by sequential study of metabolic imaging by ^13^C-MRI and EPR oximetry in two different tumor cell lines, SCCVII and HT29, grown as tumors in mice. In addition, the alteration of metabolic profile and pO_2_ status in response to 5-Gy irradiation in HT29 tumor was examined in the same manner. These results suggested that multimodal imaging would serve as a non-invasive means of studying tumor physiology and metabolism to comprehensively monitor the tumor microenvironment and associated changes in response to treatment.

## RESULTS

### Validation of serial imaging of pO_2_ and ^13^C-MRI

**Figure 1:**
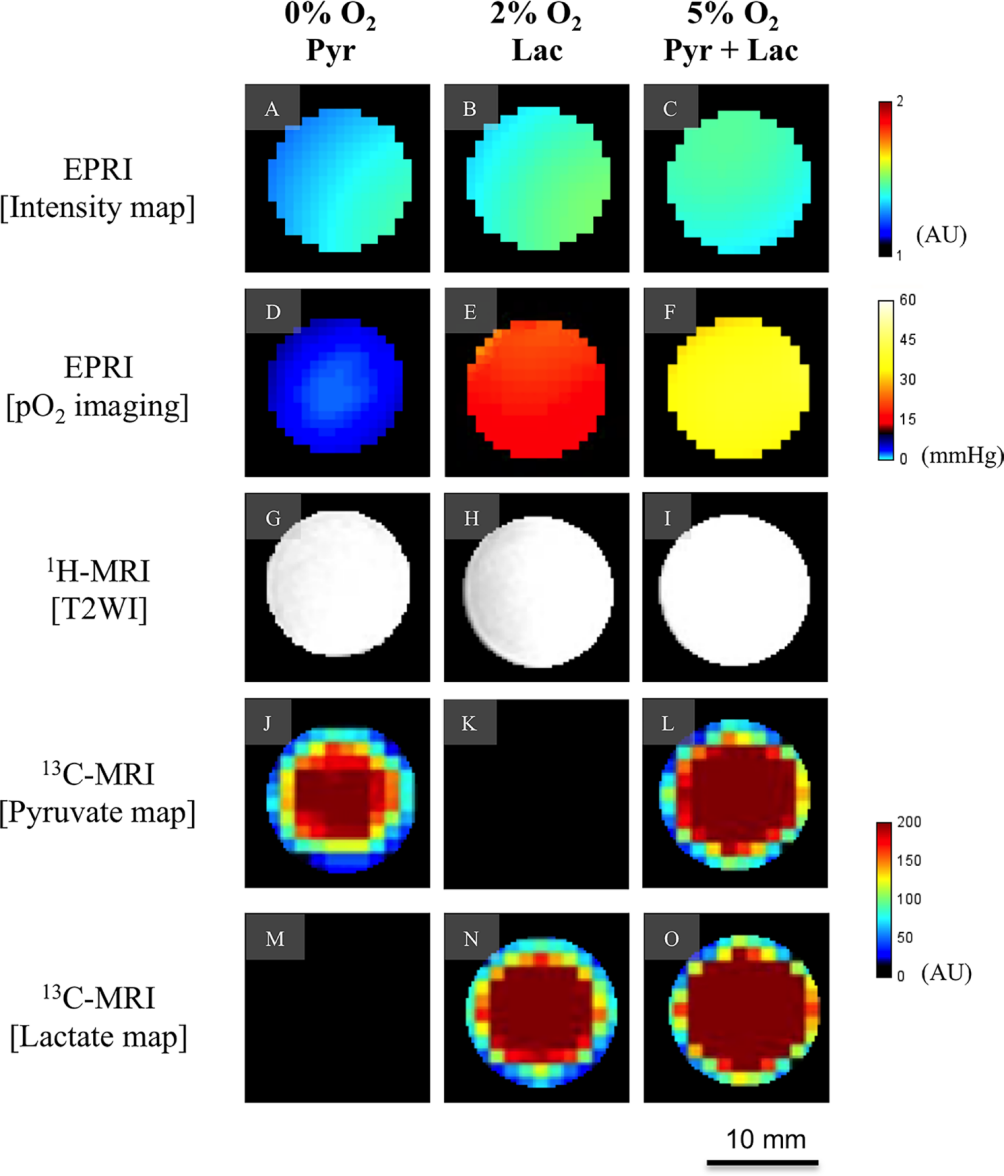
Validation of EPR oxygen imaging and [1-13C] Metabolic MRI in phantom A phantom object with three-tube of aqueous solutions containing 3 mM of the oxygen sensitive paramagnetic tracer OX063 equilibrated with 0% oxygen and 1 M [1-13C] pyruvate (left columun), 2% oxygen and 1 M [1-13C] lactate (middle column), and 5% oxygen and 1 M [1-13C] pyruvate and 1 M [1-13C] lactate (right column). Each row shows signal intensity map on EPRI (**A–C**), pO_2_ map on EPRI (**D–F**), T2-weighted image on ^1^H-MRI (**G–I**), pyruvate map on ^13^C-MRI (**J–L**), and lactate map on ^13^C-MRI (**M–O**), respectively. Color-coded indicators are shown on the right for EPRI and ^13^C-MRI.

To validate the ability of EPRI to provide images of absolute oxygen concentration and distinguish various levels of pO_2_, tubes filled with identical concentration of the paramagnetic probe, OX063 equilibrated with different levels of oxygen was studied. Figure 1A–1C show the intensity maps from the EPRI representing the concentration of OX063 and Figure 1D–1F show the corresponding maps of pO_2_. From the images, it can be seen that EPRI generated maps of pO_2_ validly represent the oxygen concentration in the solutions. T2 weighted MRI images and ^13^C images of tubes filled with aqueous solutions of pyruvate alone (Figure 1G, 1J, 1M), lactate alone (Figure 1H, 1K, 1N) and pyruvate + lactate (Figure 1I, 1L, 1O) show that it is possible to distinguish pyruvate and lactate and the combination using ^13^C-MRI. The phantom study supports the capability of the sequential image of EPR and ^13^C-MRI to monitor pO_2_ and metabolism.

### *In vivo* study

**Figure 2:**
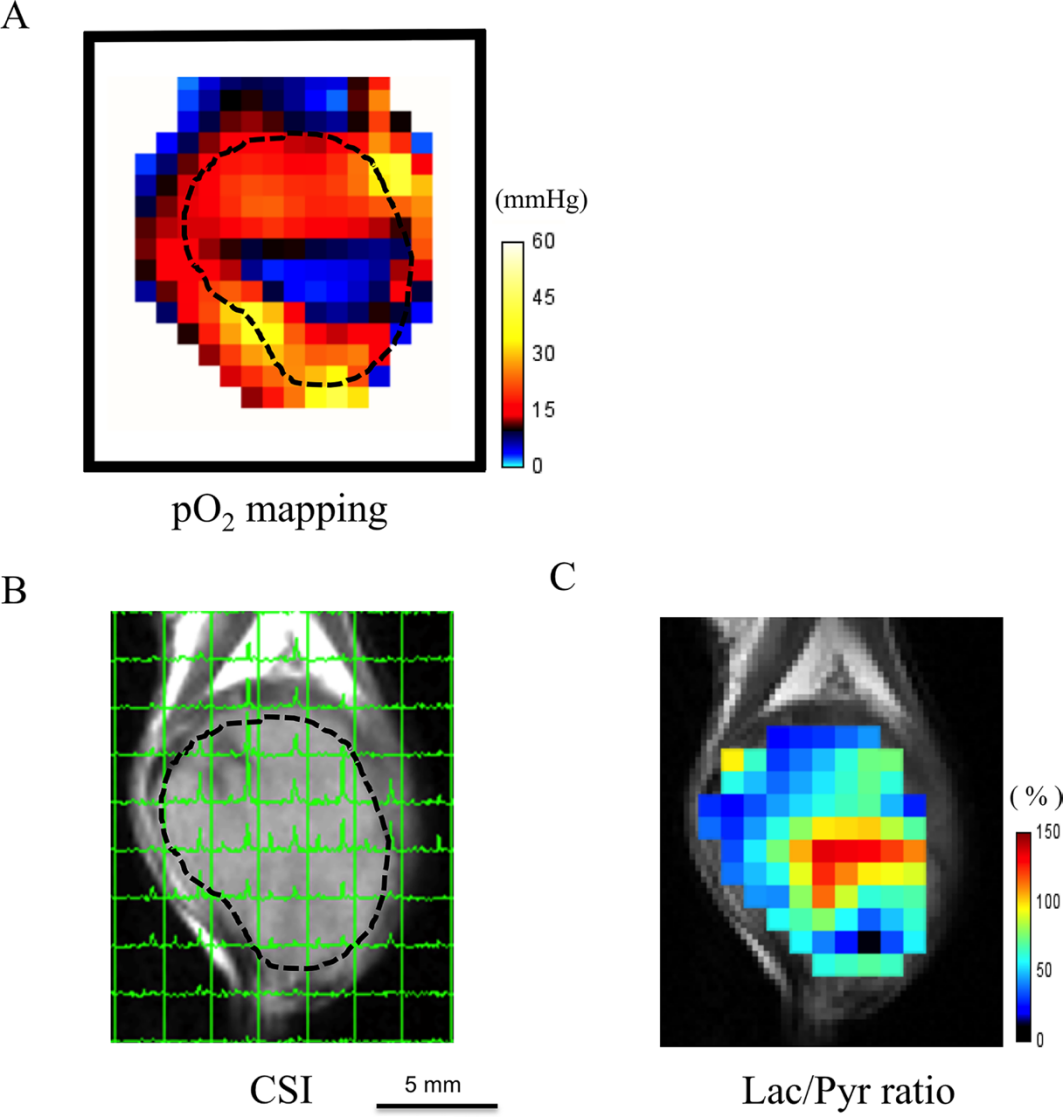
Feasibility of co-imaging of EPR oxygen imaging and ^13^C-MRI *in vivo* An example case of co-imaging of tissue EPRI pO_2_ mapping and ^13^C-MRI imaging in a mouse bearing subcutaneously SCCVII tumor on the leg. (**A**) The absolute pO_2_ map shows a hypoxic core (blue) in the central part of the tumor. (**B**) The ^13^C-chemical shift imaging (CSI) obtained 12 sec after hyperpolarized [1-^13^C] pyruvate injection overlaid on the T2-weighted image. Each signal peak corresponds to [1-^13^C] pyruvate (right) and [1-^13^C] lactate (left), respectively. (**C**) Lac/Pyr ratio map. Note that the higher lactate-to-pyruvate ratio area dominantly exists in the hypoxic region on EPRI.

The feasibility of co-imaging of tissue pO_2_ and metabolic ^13^C MRI *in vivo* was evaluated in a mouse implanted with SCCVII tumor on the leg. The absolute pO_2_ map from EPRI of the SCCVII tumor clearly shows a hypoxic region in the center of the tumor (Figure 2A, blue color) surrounded by a normoxic rim. After EPRI acquisition, the same mouse was transferred to a 7 T MRI for anatomic and metabolic imaging. Figure 2B shows the anatomic image of the tumor overlaid with ^13^C spectra obtained 12 sec after hyperpolarized [1-^13^C] pyruvate injection. [1-^13^C] Pyruvate (right peak in the spectra) and [1-^13^C] lactate (left peak) were detectable in each of tumor regions. A lactate-to-pyruvate ratio map (Figure 2C) calculated from the ^13^C spectroscopic images showed heterogeneous distribution of these metabolites within the tumor regions and relatively high lactate/pyruvate ratio was observed in the areas spatially overlapped with hypoxic regions. (Figure 2A and 2C).

### Comparison of tumor pO_2_ and pyruvate metabolism in two cancer cell lines

**Figure 3:**
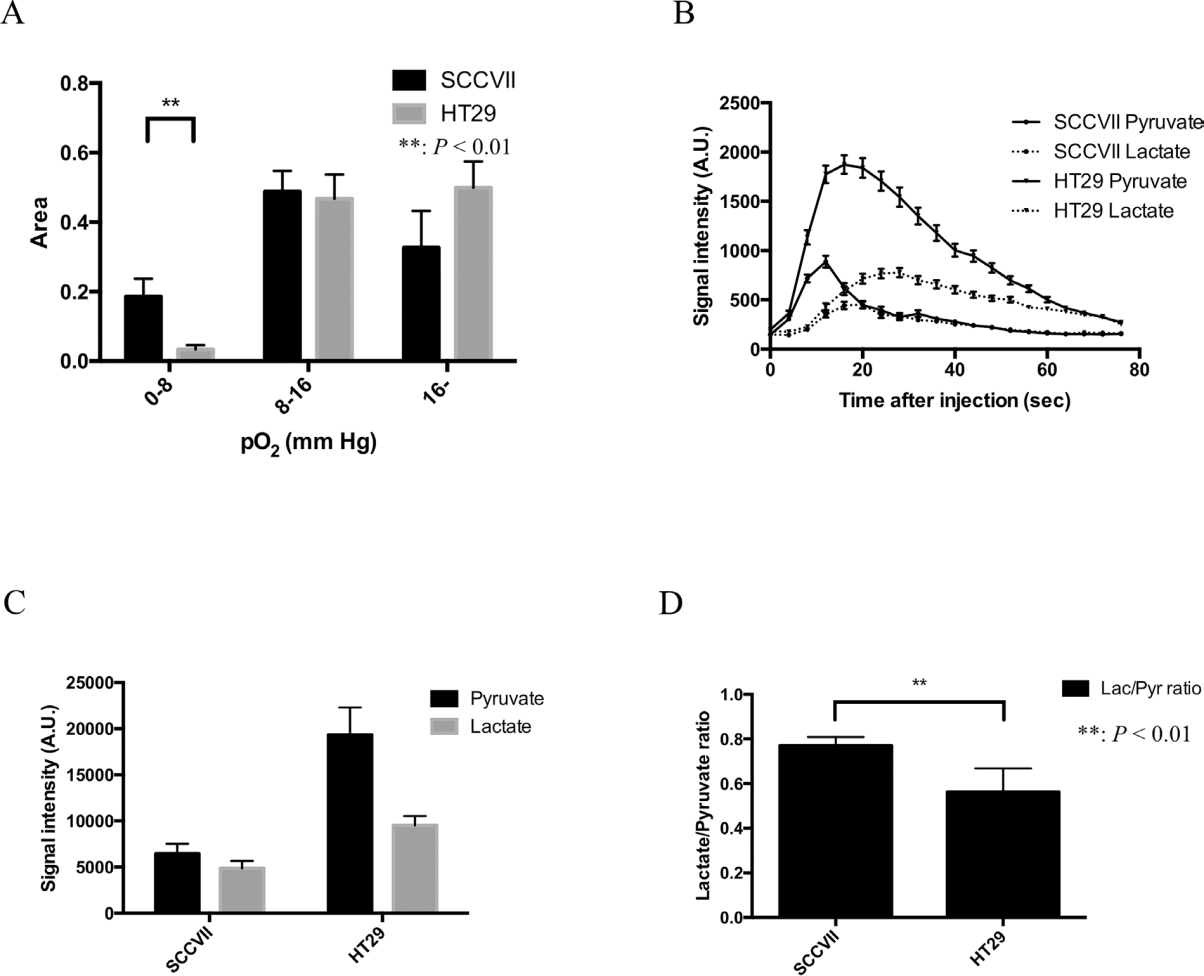
Tumor volume, pO_2_, pO_2_ fraction, and Glycolytic metabolism in SCCVII and HT29 tumor (**A**) The difference in three pO_2_ fractional areas (0–8, 8–16 and >16 mmHg) between SCCVII (*n* = 4) and HT29 (*n* = 6) tumors. (**B**) The time courses of ^13^C-MR signal intensity of [1-^13^C] pyruvate and its metabolic product [1-^13^C] lactate in SCCVII and HT29 tumor. (**C**) The sum of pyruvate and lactate signal intensity calculated from 20 images acquired during the first 80 seconds after an injection of [1-^13^C] pyruvate. (**D**). Lactate-to-pyruvate ratio in SCCVII calculated from a time course from 20 to 40 seconds. Statistically significant differences are indicated with asterisks.

Typically, the tumor size largely influences the tumor physiology and the outcome of cancer therapy. Previous studies reported that tumor oxygenation and vascularity decreased with an increase of tumor size [22, 23]. Therefore, the tumor oxygenation and pyruvate metabolism in two cancer cell lines were compared when they were almost the same in size. The tumor size of SCCVII (*n* = 4) was 643 ± 37 (mean ± SEM) mm^3^ and that of HT29 tumor (*n* = 6) was 627 ± 17 mm^3^. Although the median tumor pO_2_ of SCCVII was 13.5 mmHg, lower than that of the HT29 tumor (15.8 mmHg), the difference was not significant. Detailed analyses of pO_2_ fractions revealed that the hypoxic regions with pO_2_ < 8 mmHg in SCCVII tumor was 18.5% which was significantly larger than that of the HT29 tumor (3.3%, *P* < 0.01). On the other hand, there was no significant difference between the fraction of normoxic region with pO_2_ > 16 mmHg in SCCVII and HT29 tumor (32.6% and 49.9%, respectively) (Figure 3A). These results are consistent with previous findings from histological data for hypoxia using pimonidazole [9].

Figure 3B shows a typical time course of ^13^C-MR spectroscopy obtained after the EPRI of [1-^13^C] pyruvate and its metabolic product [1-^13^C] lactate in SCCVII and HT29 tumor. A strong lactate signal buildup was observed immediately in both tumors after the pyruvate signal in tumor region, suggesting that intravenously injected pyruvate was taken up into the cells and enzymatically converted into lactate in the tumors. Hence, the time to peak of lactate signal intensity was observed later than that of pyruvate signal both in SCCVII (Supplementary Figure 1) and HT29 tumor (Supplementary Figure 2). Figure 3C shows [1-^13^C] pyruvate and [1-^13^C] lactate signal intensity in SCCVII and HT29 tumor calculated from area under curve of the time-intensity profile of the dynamic MR spectroscopy obtained between 0 and 80 seconds. Total pyruvate in HT29 tumor was higher than that of SCCVII tumor. The total signal of pyruvate in HT29 tumor was significantly higher than lactate, whereas there was no significant difference in SCCVII tumor. On the other hand, lactate-to-pyruvate ratio calculated from the signal intensity between the 20 and 40 second in SCCVII and HT29 tumor showed that it was significantly higher in SCCVII than HT29 (Figure 3D). As a previous report form Yasui *et al*. [9] showed a remarkable fluctuation of pO_2_ within a few minutes in the SCCVII compared to HT29, where it was shown more stable. Since the main aim of our study was co-imaging of tumor hypoxia and metabolism, we selected only HT29 for co-imaging study using EPRI and 13C-MRI under a hypothesis that the oxygen status in the tumor was almost the same between the time points when the two imaging were performed.

### Hypoxic tumor sub-region increases post radiation

**Figure 4:**
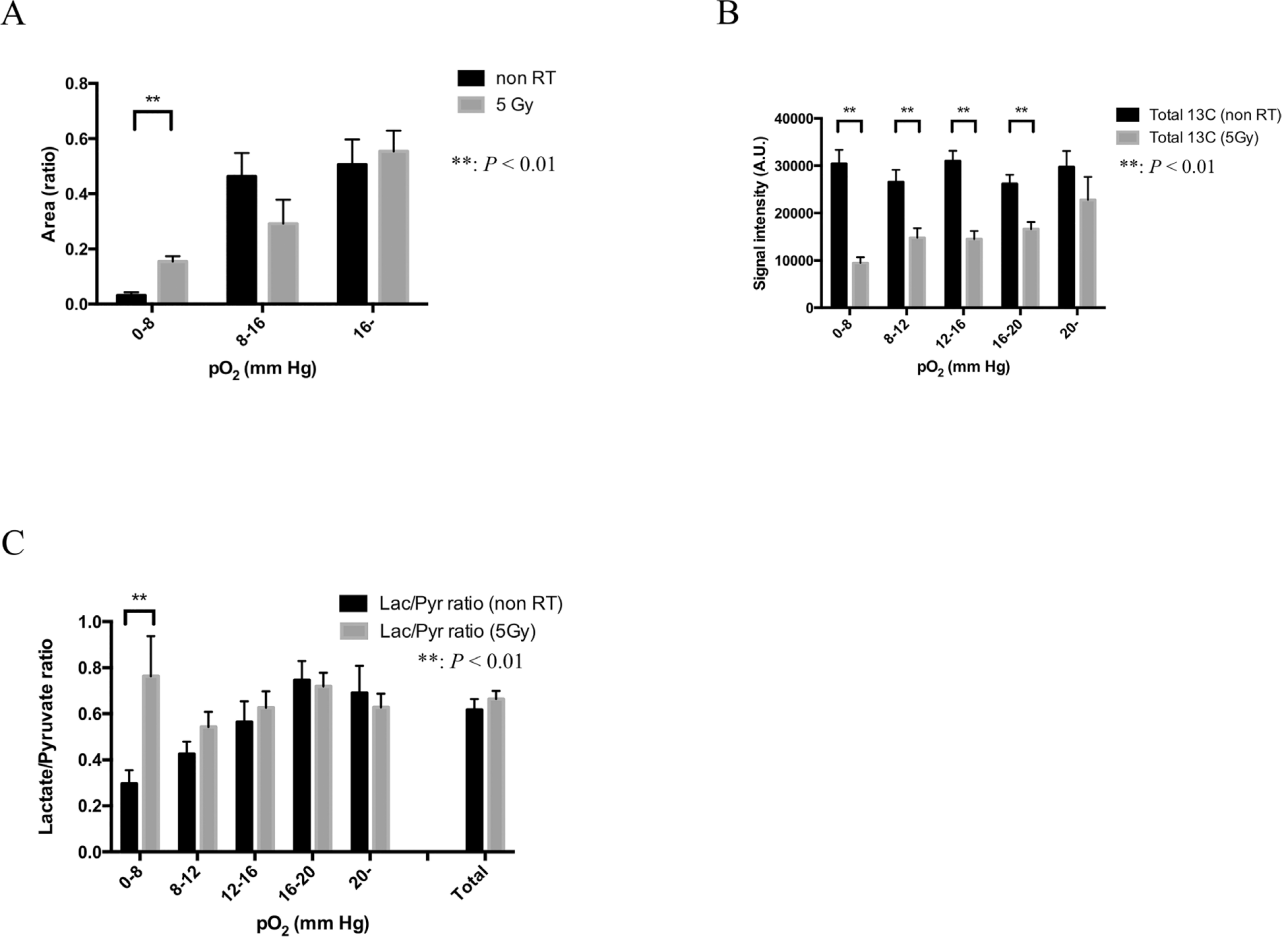
Glycolytic metabolism in each pO_2_ sub-region: comparison between non-irradiation control and 24 hours after 5-Gy irradiation in HT29 tumor (**A**) The difference in three pO_2_ fractional areas (0–8, 8–16 and >16 mmHg) between 5-Gy irradiated group (*n* = 6) and control group (*n* = 6). (**B**) The sum of the total carbon ([1-^13^C] pyruvate and [1-^13^C] lactate) signal intensity calculated from 20 images acquired during the first 80 seconds after an injection of [1-^13^C] pyruvate in five different pO_2_ fractional areas (0–8, 8–12, 12–16, 16–20 and >20 mmHg) in the two groups. (**C**). Lactate-to-pyruvate ratio calculated from a time course from 20 to 40 seconds in non-irradiation control group and 24 hours after 5-Gy irradiated group. Statistically significant differences are indicated with asterisks.

The effect of 5 Gy irradiation on tumor oxygenation was investigated by EPR oxygen imaging. Averaged tumor size in the 5 Gy-treated group (*n* = 6) was 639 ± 26 mm^3^ (mean ± SEM) and there was no significant difference statistically from that of non-treated control group (627 ± 17 mm^3^, *n* = 6). Although there was no significant difference in averaged pO_2_ over the whole tumor regions between non-treated (15.8 mmHg) and the 5 Gy-treated groups (15.2 mmHg), the fractional hypoxic volume (pO_2_ < 8 mmHg) in the 5 Gy-treated group (15.4%) was significantly higher than non-treated control group (3.2%). The fractional normoxic volume (with pO_2_ > 16 mmHg) of non-treated and the 5-Gy treated groups were 50.5% and 55.3%, respectively (Figure 4A).

### 5 Gy irradiation suppresses tumor perfusion and enhances flux of pyruvate-to-lactate conversion in hypoxic tumor sub-regions

Outcome of radiation therapy is known to depend on tumor oxygen status [2]. However post-radiation changes in the tumor metabolism are not known. We investigated the effect of a single 5-Gy irradiation on the relationship between tumor pO_2_ and energy metabolism by sequential imaging with EPRI and hyperpolarized ^13^C-MRI. Supplementary Figure 2 shows time-to-intensity curves of ^13^C-lactate and pyruvate in non-treated control and 24 hours after 5-Gy X-irradiated groups of mice bearing HT29 tumor, grouped into five sub-regions with different pO_2_ (<8, 8–12, 12–16, 16–20, and >20 mmHg). The time to peak intensity of pyruvate signal was almost the same in all the sub-regions in both non-treated and the 5-Gy irradiated groups, whereas that of lactate signal was 20 and 30 seconds in non-treated and the 5-Gy treated groups, respectively. There was no obvious pO_2_ dependency observed in these time courses of pyruvate and lactate signal intensity in non-treated control tumors, however, the difference of a time course of these signal intensity was clearly observed in the 5-Gy treated group dependent on their pO_2_ levels.

Figure 4B shows total carbon (^13^C-pyruvate + ^13^C-lactate) signal intensity calculated from the time-to-intensity curve from 0 to 80 seconds, a total of 20 images in non-irradiated 24 hours after 5-Gy irradiated HT29 tumor. The total carbon signal at 24 hours after 5-Gy irradiation of HT29 tumor was significantly lower than that of non-treated and 24 hours after 5-Gy irradiation (0–20 mmHg). Difference in the total carbon between non-treated control and 24 hours after 5-Gy irradiated tumors was largest in hypoxic tumor regions with pO_2_ < 8 mmHg, and smallest in normoxic sub-regions with pO_2_ > 20 mmHg. The decrease in total ^13^C signal in the treated group implies decrease in tumor blood perfusion, which was further confirmed by decrease in Gd-DTPA uptake during the first 1 min after injection in DCE-MRI study (Supplementary Figure 3).

Figure 4C shows lactate-to-pyruvate ratio calculated from the time-to-intensity curve from 20 to 40 seconds in non-irradiated and 24 hours after a 5-Gy irradiated HT29 tumor. Lactate-to-pyruvate ratio in 0–8 mmHg at 24 hours after 5-Gy irradiation of HT29 tumor (76%) is higher (*P* < 0.01) than of non irradiation of HT29 tumor (36%), suggesting that the enzymatic conversion of pyruvate to lactate was much higher 24 hours after 5-Gy irradiation in the area with 0-8 mmHg, especially under 16 mmHg.

### *In vitro* study

#### Extracellular acidification rate (ECAR) as a maker of lactate production

**Figure 5:**
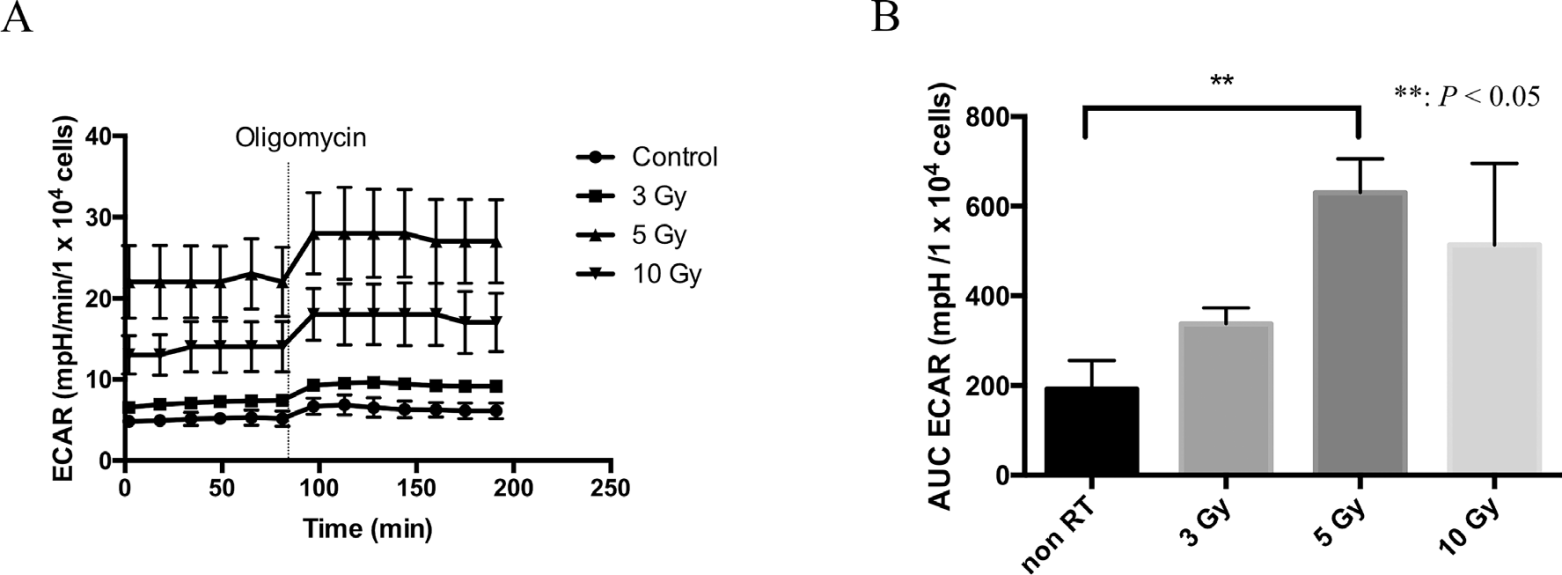
Extracellular acidification rate (ECAR) as a marker of lactate production (**A**) The time course of ECAR in control, 3-, 5-, and 10-Gy irradiated cells in tissue culture. (**B**) The area under curve (AUC) of ECAR corrected by the cell numbers after an administration of oligomycin. Statistically significant differences are indicated with asterisks.

To confirm the increase in lactate-to-pyruvate ratio after irradiation on ^13^C-MRI, we conducted an *in vitro* study independently, where lactate production form the tumor cells were assessed. In this experiment, subcutaneous HT29 tumors were excised 24 h after 0, 3, 5, or 10-Gy irradiation followed by homogenization and trypsin-incubation to prepare single cells. Using 1 × 10^4^ cells in each well of a 96-well plate, extracellular acidification rate (ECAR) was measured by SeaHorse extracellular flux analyzer as a maker of lactate production. Figure 5A shows a time course of ECAR in the culture media with the cells treated by 0, 3, 5, or 10 Gy and Figure 5B shows AUCs of ECAR. A significant difference between the increase in ECAR in the non-irradiated control group and that in the 5-Gy irradiation group (192.0 vs 630.3 mpH/10^4^ cells) was observed after an injection of ATP synthase inhibitor, oligomycin. The increase in ECAR of 10-Gy treated cells was smaller than 5-Gy treated cells presumably as a result of substantial damage of the cells.

## DISCUSSION

Technological developments in medical physics for the precise delivery of radiation dose has been paralleled in recent years by a greatly-improved molecular imaging techniques which can profile the tumor microenvironment in terms of physiology band metabolism. To take full advantage of the curative potential of modern and modern radiotherapy techniques such as stereotactic radiotherapy, radiosurgery, and intensity-modulated radiotherapy (IMRT), molecular imaging to profile the microenvironment is needed for identification of target volumes [24–28]. Recently, the development of these technologies has been drawing clinicians’ attention to molecular imaging for prospect of highly targeted radiation therapy and providing new research opportunities in preclinical and clinical study in small-animal models. Tumor blood flow is one of the important biological parameters in the cancer therapy because it would affect not only drug distribution but also tumor hypoxia as a result of impaired blood flow that may cause radiation resistance. Therefore, molecular imaging techniques which enables us to assess the tumor metabolism and oxygenation state before and during its treatment is needed [23]. In this study, we utilized a system where the oxygen mapping and the metabolic imaging to be co-registered using EPRI and hyperpolarized ^13^C-MRI and successfully showed correlation between the oxygen partial pressure and lactate-pyruvate distribution on the anatomical images. First, we validated the feasibility of this method using *in vitro* and *in vivo* (Figures 1 and 2). Figure 2A and Figure 2C showed the heterogeneous oxygen distribution and lactate-to-pyruvate ratio, respectively, in the same representative SCCVII tumor. They also described the regions with high lactate-to-pyruvate ratio spatially concordant with the regions with hypoxia. The differences in tumor hypoxic state and pyruvate metabolism between SCCVII and HT29 tumor were clearly observed. Since majority of signal peaks observed immediately after the pyruvate signal peak on hyperpolarized ^13^C-MRI was from lactate, we also estimated the blood flow volume using a summation of peak signal intensity of pyruvate and lactate as the approximate total signal from carbon-13. There was a significant difference in the signal intensity from total carbon between SCCVII and HT29 tumor, suggesting lower perfusion in SCCVII tumor than HT29 (Figure 3C). The longer time-to-peak both for pyruvate and lactate after injection in SCCVII was consistent with this observation (Figure 3B). Therefore, the higher lactate-to-pyruvate ratio observed in SCCVII than HT29 as shown in Figure 3D was consistent with higher hypoxic fraction (pO2 < 8 mmHg) in SCCVII presumably due to higher LDH activity under anaerobic glycolytic state (Figure 3A). Yasui *et al*. [9] showed that the number of pericytes that cover blood vessels within SCCVII tumors was relatively small compared with the HT29 tumors and this difference could contribute to the difference not only in blood perfusion but also in O2 diffusion. As Park *et al*. [29] had shown decrease in tumor blood perfusion after radiation therapy, we developed a co-imaging technique to investigate changes in oxygen distribution and glycolytic metabolism modifications as a result of radiation exposure using HT29 tumor, which Yasui *et al*. [9] had reported relatively stable in fluctuation in oxygen status compared to SCCVII. A dynamic contrast enhanced MRI study using gadolinium contrast media is one of the well-known methods for analyses of blood perfusion in tumors and has been used to measure several relevant parameters, the microvasculature volume, vascular permeability and blood flow in various tissues [30, 31]. We confirmed that gadolinium concentration in the early phase, which reflected blood perfusion, at 24 hours after 5-Gy irradiation in HT29 tumor was lower than non-RT group (Supplementary Figure 3). This result suggested a decrease in blood flow volume resulting radiation treatment. An increase in hypoxic area (pO_2_ < 8 mmHg) by over 5-fold was observed 24 hours after 5-Gy irradiation (Figure 4A) whereas a significant decrease in total carbon-13 signal was observed not only in hypoxic area but also in the more oxygenated areas (8–20 mmHg) (Figure 4B), indicating the radiation-induced hypoxia was attributed to a global decrease in the blood flow. It is notable that there was no significant difference in total carbon-13 signal before irradiation among all of the areas including hypoxic area. This is probably because the hypoxic region in HT29 tumor before irradiation was so small that carbon-13 signals from this area could be buried with those from the surrounding more oxygenated tissue because of the thickness of FOV in ^13^C-MRI larger than EPRI (8 mm vs. 2 mm, respectively). Likewise, a significant increase in the lactate-to-pyruvate ratio by more than 2-fold observed in the hypoxic area 24 hours after irradiation could be at least partially explained by hypoxic regions newly developed by radiation-induced hypoperfusion area where anaerobic glycolysis dominates grew large enough to bring the ^13^C signal detectable.

HIF-1α, which is known to be stabilized when O_2_ availability is decreased to regulate transcription of hundreds of genes that encode proteins including pyruvate dehydrogenase kinase-1 (PDK1) and lactate dehydrogenase (LDH) [32–35]. Phosphorylation of pyruvate dehydrogenase by PDK1 reduces flux through the TCA cycle, as a result, down-regulates aerobic respiration. Pyruvate accumulated in hypoxic cells is then converted into lactate via LDH [36]. In our *ex vivo* experiment, enhanced glycolytic reserve was indicated by a significant increase in ECAR after irradiation (Figure 5). These measurements were performed at relatively higher and uniform oxygen (21%) conditions in contrast to solid tumors. Kim *et al*. [33] reported hypoxia-independent stabilization of HIF-1a by a fractionated 6-Gy irradiation in glioma cell lines, suggesting there could be radiation-induced metabolic change independent on alteration of blood perfusion. Combining these findings, although still speculative, the increase in pyruvate-to-lactate ratio in the hypoxic region observed in ^13^C-MRI after irradiation might be also attributed to radiation-induced HIF-1a stabilization in addition to hypoperfusion.

In this study, we successfully showed the feasibility of sequential imaging of the pO_2_ map using EPRI in combination with a hyperpolarized ^13^C-MRI that provided valuable metabolic information including dynamic flux of pyruvate-to-lactate conversion. A limitation in this study is insufficient spatial resolution of ^13^C-MRI that could pass over small hypoxic lesions detected on EPRI. Since it needs 8 - 10 mm slab thickness to acquire sufficient MR signals from hyperpolarized carbon-13 while the in-plane resolution is 2 mm. Nevertheless, developing state-of-the-art imaging methods, such as MRI and PET in combination with technical improvements in hypoxic and metabolic imaging technique based on our study would contribute to further improvement in treatment outcome by developing strategies of a highly personalized, tailored treatment utilizing targeting therapy against hypoxia and subsequent metabolism changes in the tumor using [37]. Radiation therapy alters the pO_2_ distribution in the tumor. The co-imaging of pO_2_ and metabolic imaging of pyruvate-to-lactate conversion kinetics can shed light on the local metabolic changes especially in hypoxic area induced by radiation therapy.

## MATERIALS AND METHODS

### Ethics statement

We carried out all our procedures in compliance with the Guide for the Care and Use of Laboratory Animal Resources (National Research Council, 1996), and experimental protocols were approved by the National Cancer Institute Animal Care and Use Committee (NCI-CCR-ACUC (Bethesda, USA), Protocol# RBB- 159).

### Phantom model

Since one of the purposes of this experiment was to assess the feasibility of serial imaging of pO_2_ and [1-^13^C] using EPRI and MRI we conducted a phantom study first. Three identical cylinder-shaped glass container filled with ultrapure water containing 3 mM of oxygen-sensitive tracer, triarylmethyl (TAM; methyl-tris[8-carboxy-2,2,6,6-tetrakis[(2-hydroxyethyl]-benzo[1,2-d:4,5-d′]bis[1,3]dithiol-4-yl] trisodium salt; OX063, GE Healthcare) were used. Each solution was equilibrated with (1) 0% oxygen and 1 M [1-^13^C] pyruvate, (2) 2% oxygen and 1 M [1-^13^C] lactate, and (3) 5% oxygen, 1 M [1-^13^C] pyruvate and 1 M [1-^13^C] lactate, respectively, for both pO_2_ imaging by EPRI and ^13^C-MR spectroscopy along with anatomical imaging by 7T MRI.

### Cell culture and tumor implantation

Murine squamous cell carcinoma SCCVII and human colon cancer HT29 cell lines were tested in April 2013 by IDEXX RADIL (Columbia, MO) using a panel of microsatellite markers and authenticated. Female C3H/Hen mice and athymic nude mice were supplied by the Frederick National Laboratory for Cancer Research Center (Frederick, MD). SCCVII and HT29 solid tumors were formed by a subcutaneous injection of 5 × 10^5^ cells and 1 × 10^6^ cells, respectively, into the right hind legs of mice as described previously [8]. The experiments were initiated when tumors grew to approximately 600–700 mm^3^. The tumor volume was calculated by a following approximation formula; Tumor volume = length × width × height × 3.14 × 1/6. The body weight measured before the experiments was ranged from 21 to 27 g. In the imaging of EPRI and MRI, mice were anesthetized by isoflurane inhalation (4% for induction and 1.5% for maintaining anesthesia) in medical air (750 mL/min) and positioned prone with their tumor-bearing legs placed inside the resonator. During EPRI and MRI, the breathing rate of the mouse was monitored with a pressure transducer (SA, Instruments Inc.) and maintained at 60 ± 10 breaths per minute. Core body temperature was monitored by a FISO FTI-10 temperature sensor (FISO Technologies Inc., Quebec, Canada) and maintained at 37° C with a flow of warm air (EPRI) or water (MRI). For administration of OX063 and [1-^13^C] pyruvate solution, a 30-gauge needle was cannulated into the tail vein and extended using a polyethylene tubing.

### EPR imaging for pO_2_

Technical details of the EPR scanner and oxygen image reconstruction were described in earlier reports [8, 11, 38–40]. Parallel coil resonators tuned to 300 MHz were used for EPRI and MRI because the use of radiofrequency (RF) at 300 MHz for EPR allows the EPR signal to be acquired deeper in the tissue of a living object. After the animal was placed in the resonator, the resonator (17 mm in diameter. and 17 mm long) was used as an identical coil for EPRI and MRI operating at 300 MHz. For EPRI experiment, OX063 was injected intravenously through a cannula placed in the tail vein. To keep the blood concentration, OX063 was given as a 1.125 mmol/kg bolus injection followed by 0.04 mmol/kg/min continuous injection [41]. EPR signals were collected following the radiofrequency excitation pulses; 60 ns, 80 W, 70° flip angle using an analog digital converter (200 M samples/s). EPR measurements were started 3 min after OX063 injection, and it took 12 min to obtain a data set for a 3D image. Although the in-plane spatial resolution at the acquisition was 1.8 mm, it was digitally enhanced subsequently to 0.125 mm for co-registration with MRI images. Since Saito *et al*. [42] showed that a transient decrease in tumor oxygenation after intravenous administration of pyruvate, we underwent EPRI before ^13^C imaging.

### MRI and co-registration of pO_2_ images with anatomic images

After each EPRI scan, the mouse was transferred into a 7T MRI scanner without being removed from the holder to obtain the T2-weighted anatomical image. Anatomical images of the tumor-bearing leg were obtained using 7T MRI scanner controlled with ParaVision 5.0 (Bruker BioSpin MRI GmbH, Billerica, MA). After a quick assessment of the sample position by a fast low-angle shot (FLASH) pilot sequence, T_2_-weighted axial and coronal images were obtained using a fast spin echo sequence (RARE) with an echo time of 13 ms, repetition time of 2,500 ms, 16 slices, RARE factor 8, and a resolution of 0.125 × 0.125 mm^2^. For convenience of co-registration with EPRI, all MRI images had the same filed of view (FOV) of 32 mm and slice thickness of 2 mm. Co-registration of EPRI and MRI images was accomplished using a code written in MATLAB (Mathworks) script as previously described [8, 18, 43].

### DNP-MRI of [1-^13^C]-labeled pyruvate metabolism

After acquiring anatomical T_2_-weigthed MRI data set, hyperpolarized [1-^13^C] MRI studies were performed. To obtain hyperpolarized [1-^13^C] pyruvate, 30 μL of pyruvic acid (1-^13^C, 99%, Cambridge Isotope Laboratories, Tewksbury, MA) containing 15 mM of OX063 and 2.5 mM of the gadolinium chelate ProHance (Bracco Diagnostics, Milano, Italy) were polarized at 3.35 T and 1.4 K in the Hypersense DNP polarizer (Oxford Instruments, Abingdon, UK), according to the manufacturer's instructions. After 1–1.5 h, the hyperpolarized sample was rapidly dissolved in 4.5 mL of a superheated alkaline buffer comprising 40 mM of 4-(2-hydroxyethyl)-1-piperazineethanesulfonic acid, 30 mM of NaCl, and 100 mg/L of ethylendiaminetetraacetic acid to obtain 96 mM [1-^13^C] pyruvate solution. Appropriate quantity of NaOH was added to the dissolution buffer to adjust the pyruvate solution to be pH 7.4. Immediately after the dissolution, a bolus injection of 300 μL (approximately 1.15 mmol/kg) [1-^13^C] pyruvate solution was administrated intravenously through the tail vein cannula right after the dissolution [18]. For the dynamic study, ^13^C spectra were acquired every 4 seconds using echo-planar spectroscopic imaging technique after the start of pyruvate injection from a 8- to 12-mm slice on the SCCVII and HT-29 for 240 seconds. The slice thickness was defined as it included most part of the tumor. ^13^C two-dimensional spectroscopic images were also acquired 30 seconds after the start of the pyruvate injection with a 32 × 32 mm^2^ FOV in a 8 mm slice of horizontal plane through the longitudinal axis of the leg a matrix size of 16 × 16, spectral width of 9765.62 Hz, repetition time of 125 ms. The total time required to acquire each image was 19.2 seconds.

### Gadolinium enhanced MRI

For T1 mapping, coronal RARE images of four slices passing through the tumor region were obtained with TR values of 300, 2000, and 6000 ms. FLASH sequence of the same four slices was applied for dynamic contrast enhanced (DCE)-MRI using an administration of contrast agent, gadolinium (Gd)-DTPA (Magnevist, Beyer Pharmaceuticals, Berlin, Germany). The scan parameters were as follows: TE 4.00 ms, TR 156 ms, flip angle 45°, three slices, 0.50 × 0.50 mm^2^ in-plane resolution, 20-second acquisition time per each dynamic scan, and 98 times repetition. Gd-DTPA (50 mM, 5 μL/g body weight) was intravenously injected into the tail vein 2 min after the beginning of the fast gradient echo scan. The gadolinium concentration in the tumor tissue was calculated as described in a previous report [44].

### Extracellular acidification rate (ECAR)

HT29 tumor mice were divided into four groups (control, 3, 5, and 10 Gy-irradiation, *n* = 3 each group). The mice were euthanized by breathing carbon dioxide gas 24 hours after an irradiation. Tumor tissues were immediately excised and minced by scissors and digested by 0.2% collagenase/0.02% deoxyribonuclease solution to be single-cell suspensions. The XF96 Extracellular Flux Analyzer (Seahorse Bioscience, North Billerica, MA) was used to detect rapid, real-time changes in cellular glycolysis rate. Extracellular acidification rate (ECAR) was then measured according to the recommended protocol provided by the manufacturer [45]. Before measurements, 20,000 cells were cultured 6 hours in custom XF96 microplates and they were immersed in 200 μL of unbuffered medium after being washed with the medium once, followed by incubation in the absence of CO_2_ for 1 h. After measuring basal ECAR, oligomycin were introduced in real time. Because oligomycin-treated cells can only produce ATP via glycolysis, oligomycin provides a measure of maximum glycolytic potential. Analysis of ECAR reflects lactate excretion and serves as an indirect measure of glycolysis rate. Data are expressed as the mean ± SEM of these values.

### X-ray irradiation

The animals were restrained without anesthesia in a custom jig to be irradiated only on the tumor-bearing leg. Tumors were irradiated 5 Gy using an X-ray irradiator, XRAD-320 (Precision X-ray Inc., North Branford, CT). Imaging examinations were performed 24 hours after irradiation.

### Statistical analysis

All results were expressed as the mean ± SEM. The differences in means of groups were determined by 2-tailed Student's *t* test. The minimum level of significance was set at *P* < 0.05.

## SUPPLEMENTARY MATERIALS AND FIGURES


