# Supplementary material for: Co-imaging of the tumor oxygenation and metabolism using electron paramagnetic resonance imaging and 13-C hyperpolarized magnetic resonance imaging before and after irradiation
Source: Oncotarget. 2018 May 18;9(38):25089–100. doi: 10.18632/oncotarget.25317 (PMC5982751; doi:10.18632/oncotarget.25317)
Supplement: Supplementary file 1 [file oncotarget-09-25089-s001.pdf]

Co-imaging of the tumor oxygenation and metabolism using electron paramagnetic resonance imaging and 13-C hyperpolarized magnetic resonance imaging before and after irradiation

SUPPLEMENTARY MATERIALS

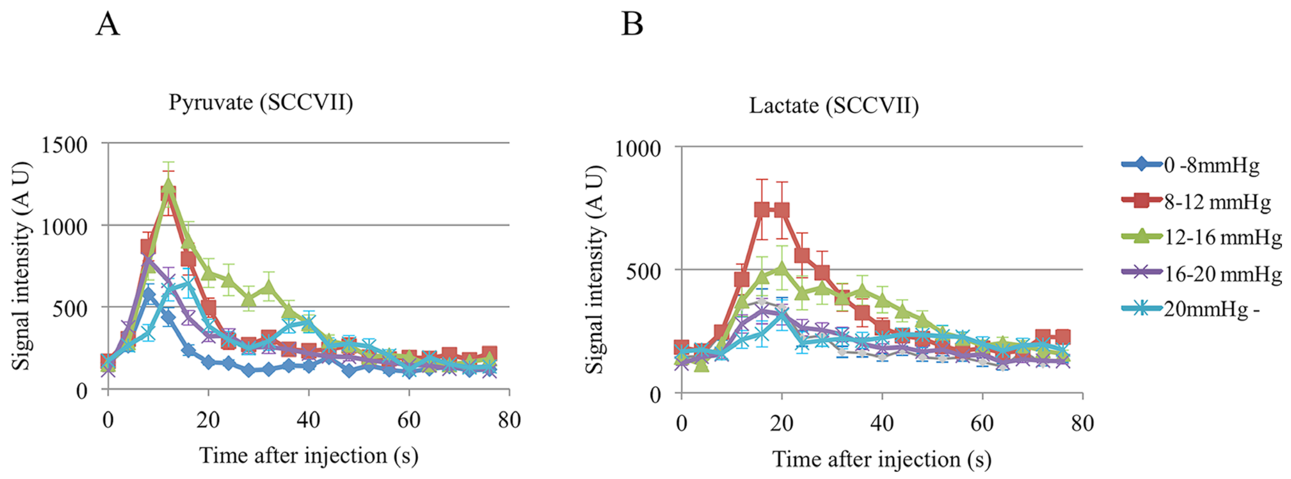

Supplementary Figure 1: The time courses of the ^{13}C-MR signal intensity of [1-^{13}C] pyruvate (**A**) and its metabolic product [1-^{13}C] lactate (**B**) in SCCVII tumor in the five different pO_2 areas (0–8, 8–12, 12–16, 16–20 and >20 mmHg).

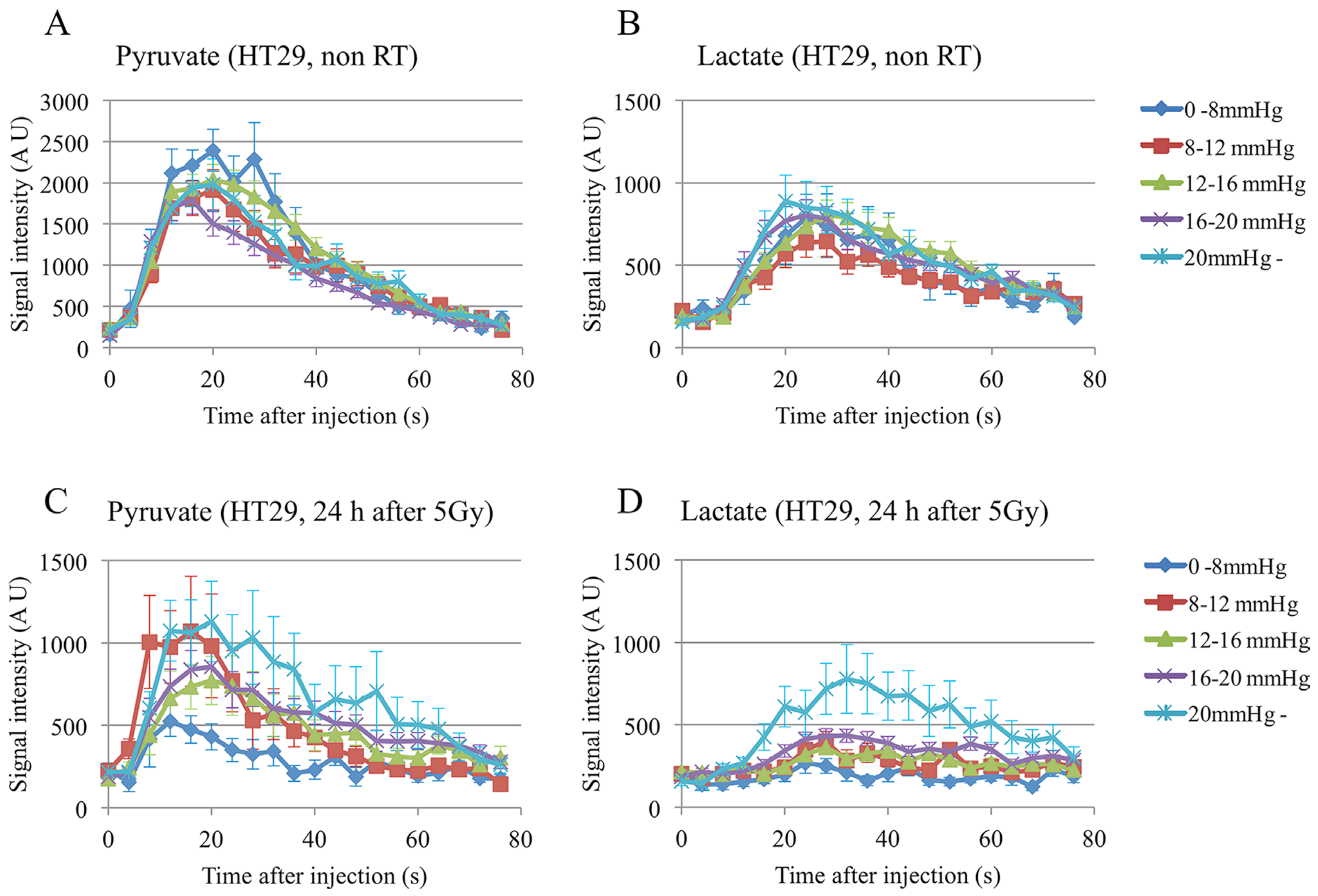

Supplementary Figure 2: The time courses of ^{13}C MR signal intensity of [1-^{13}C] pyruvate and its metabolic product [1-^{13}C] lactate in non-irradiated HT29 tumor (**A** and **B**, respectively) and 5-Gy irradiated HT29 tumor after 24 hour (**C** and **D**, respectively) in the five different pO_2 areas (0–8, 8–12, 12–16, 16–20 and >20 mmHg).

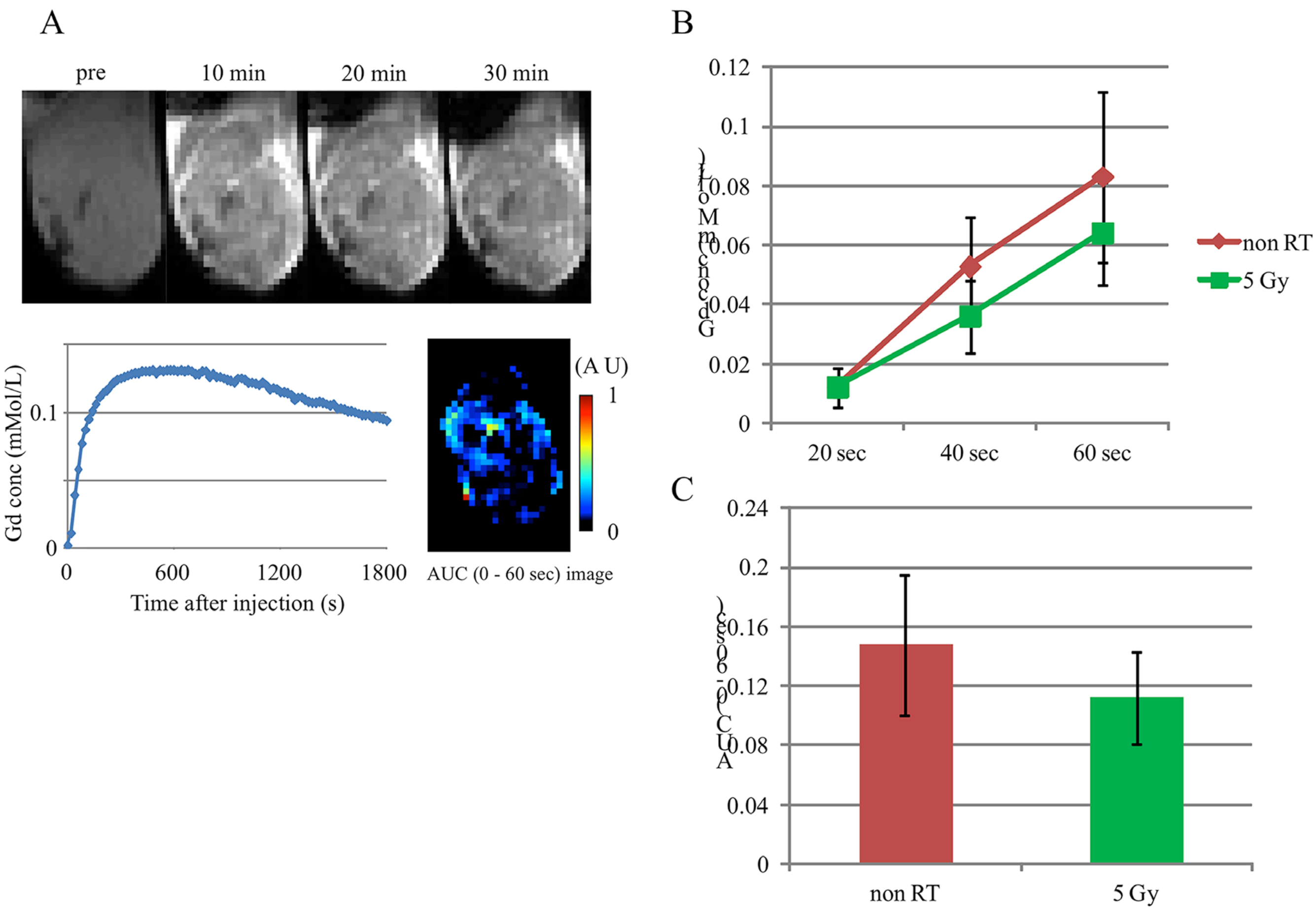

Supplementary Figure 3: The change in gadolinium concentration: comparison between non-irradiation control and 24 hours after 5-Gy irradiation in HT29 tumor. (A) A typical case of a dynamic contrast enhanced T1-weighted MR images (pre, 10, 20 and 30 minutes after an injection of gadolinium chelate) along with a dynamic curve of averaged gadolinium concentration in the tumor. **(B)** A time course of gadolinium concentration in the early phase (0–60 seconds) in non-irradiated HT29 tumors ($n = 4$) and those irradiated with 5-Gy before 24 hours ($n = 4$). **(C)** AUC of a time course of gadolinium concentration from 0 to 60 seconds in the two groups. The averaged concentration of gadolinium in the irradiated group is significantly lower than that in non-irradiated group (0.112 mmol/L and 0.148 mmol/L, respectively; $P < 0.05$).
